# Hand Hygiene Practices in School Populations: Assessing Their Impact on Infectious Disease Outbreaks

**DOI:** 10.1111/jpc.70311

**Published:** 2026-02-10

**Authors:** Namita Singh, Alan Silburn, Nicholas Moore

**Affiliations:** ^1^ Western Sydney University Campbelltown New South Wales Australia; ^2^ Emergency Department Campbelltown Hospital Campbelltown New South Wales Australia

**Keywords:** hand hygiene, infectious disease prevention, outbreak, paediatric infectious disease, preschool, school

## Abstract

**Aim:**

This review aimed to evaluate the impact of school‐based hand hygiene interventions on infectious disease outcomes in school populations.

**Methods:**

A PROSPERO registered review (CRD42024620293) followed by Cochrane methodology MEDLINE, Embase, PubMed and ERIC formed the databases that received the search. The study included English‐language research from 2014 to the present that examined school children in educational settings with quantifiable hand hygiene‐disease relationships. The screening process and appraisal followed the Critical Appraisal Skills Programme (CASP) checklist.

**Results:**

The search produced 4345 records from which 33 studies qualified for inclusion. The interventions included educational programmes and handwashing facilities together with hand sanitiser distribution and Water, Sanitation and Hygiene (WASH) programmes that combined multiple components. Education‐only interventions reduced upper respiratory infection‐related absences by up to 50%. Soap and water handwashing combined with sanitiser reduced acute gastroenteritis absences by 36%. Integrated WASH programmes lowered diarrhoea and respiratory illness absences. Parental involvement further enhanced effectiveness.

**Conclusions:**

Hand hygiene programmes are most effective when combining education, resources and behaviour change. Their success depends on context, infrastructure and delivery. While reducing disease and absenteeism, challenges like dermatitis and non‐compliance remain. Tailored, inclusive approaches and policy‐level investment are needed to ensure sustainable, evidence‐based impact in schools.

## 1 Introduction

Paediatric infectious disease outbreaks remain a major public health concern, significantly affecting school attendance and parental productivity, with broad educational and economic implications [1]. This review assesses the impact of hand hygiene practices on infectious disease outbreaks in paediatric populations, focusing on school settings and examines behavioural differences influencing disease transmission.

## 2 Background

Despite early advancements, handwashing was not widely adopted until the 1980s, when outbreaks of foodborne illnesses and healthcare‐associated infections prompted the U.S. Centres for Disease Control and Prevention to promote it as an essential infection control measure. This led to the first national hand hygiene guidelines, inspiring similar initiatives globally [2].

One such initiative has had well‐documented success; the Five Moments for Hand Hygiene programme in hospital settings demonstrates the transformative impact of structured hand hygiene initiatives on reducing infection rates [3]. By emphasising key moments for hand hygiene: before patient contact, before aseptic tasks, after exposure to body fluids, after patient contact, and after contact with patient surroundings, this programme has significantly reduced healthcare‐associated infections worldwide. Its effectiveness inspires a model that could be adapted for school environments to improve hand hygiene practices and reduce infectious disease transmission [3].

A similar structured approach in schools, targeting moments such as before meals, after toilet use, and after play, could reduce respiratory and gastrointestinal outbreaks, which disrupt education and compromise child well‐being [4, 5]. These outbreaks remain prevalent in younger age groups, particularly school‐aged children. Globally, paediatric infectious diseases cause about 1.4 million deaths annually and result in significant productivity losses and healthcare costs, especially in low‐ and middle‐income countries [6]. While immunisations, contact precautions, and school closures help mitigate transmission, the relationship between children's hand hygiene practices and outbreak prevalence is less thoroughly documented. From this, the following PICO question was derived:

PICO Question: In paediatric populations, how do hand hygiene practices impact the prevalence of paediatric infectious disease outbreaks?

While previous reviews have examined hand hygiene and infection prevention broadly, many have focused on household or healthcare settings, preschool populations or have pooled heterogeneous age groups and contexts. This review advances existing literature by synthesising contemporary, school‐based evidence in children aged 5–18 years, explicitly examining educational, behavioural and implementation outcomes alongside infection and absenteeism measures, and by contextualising findings across World Bank income settings. In doing so, it provides a more policy‐relevant assessment of how hand hygiene interventions function within real‐world school environments.

## 3 Methods

The review followed Cochrane methodology to examine links between hand hygiene and paediatric respiratory and gastrointestinal infections, based on a preregistered PROSPERO protocol (CRD42024620293) [7]. Although the protocol included preschool and school‐aged children, this manuscript reports school‐aged results only.

### 3.1 Search Strategy

A literature search was conducted in the electronic databases MEDLINE Complete, Embase and PubMed as they cover a wide range of scientific, medical and healthcare disciplines [8]. In addition, the Education Resources Information Centre (ERIC) electronic database permitted literature from the educational perspective of disease burden, thus adequate for the proposed research question.

The keywords and Medical Subject Headings (MeSH) used during the search were ‘respiratory tract infection, common cold, influenza, Coronavirus, respiratory syncytial virus, viral gastroenteritis, salmonella, campylobacter, *Escherichia coli* , shigella and *Staphylococcus aureus* ’. Population‐defining terms used were student, school, preschool, daycare, child, children or infants. For the intervention, proximity operators were utilised to within seven words between ‘hand’ and ‘wash, disinfect, sanitise, clean or hygiene’. The search strategies used for each database are outlined in Appendix S1.

### 3.2 Eligibility Criteria

All studies retrieved from the electronic database searches were imported into EndNote X8 [9], where duplicates were identified and removed. The search strategy applied database limiters to include only literature published in English from 1 January 2014 onwards, to ensure relevance and currency.

Only studies published in English were included to ensure feasibility and consistency in screening and data extraction. This restriction may have introduced language bias, potentially excluding relevant studies published in other languages, particularly from non‐English‐speaking regions.

Studies were excluded if the link between hand hygiene and infection outcomes was unclear or unidentifiable. Additional exclusion criteria included review articles, case reports, editorials and any non‐peer‐reviewed publications. To enhance the generalisability of the findings to educational environments, studies conducted in healthcare facilities, households, communities or other non‐educational settings were also excluded. Studies involving neonates, newborns or children with confounding health conditions were likewise considered ineligible.

The screening process followed a three‐stage exclusion approach. In the first stage, article titles were reviewed to eliminate studies with non‐relevant interventions or population characteristics. In the second stage, abstracts were screened for relevance based on the same criteria. In the third stage, full‐text articles were assessed to ensure that the key variables of interest were identifiable.

Each reviewer independently screened the articles, blinded to the other's decisions. Separate inclusion lists were compiled and subsequently compared. Articles selected by both reviewers were automatically included, while those rejected by both were excluded. For articles selected by only one reviewer, further discussion and critical appraisal were undertaken using the Critical Appraisal Skills Programme (CASP) methodology to determine final inclusion [10]. This approach ensures objectivity and rigour in the selection process. The results of the CASP are given in Appendix S2.

### 3.3 Risk of Bias Assessment

In assessing the risk of bias for the included studies, 33 articles were evaluated using the CASP methodology, which was applied specifically to each study design. The CASP tool enables the systematic evaluation of methodological quality by assessing criteria such as study design, sample size, data collection methods and analysis techniques. Each article was independently assessed for potential sources of bias, such as selection bias, performance bias, detection bias and reporting bias. Discrepancies in risk assessments were discussed among the review team to reach a consensus. Various tools were used to assess the risk of bias, including the Cochrane Risk of Bias 2 (RoB2), the ROBINS‐E and ROBINS‐I tools. The data from these assessments are presented in Appendix S2, with the overall risk of bias mentioned in Table 1.

**TABLE 1:** Literature characteristics.

Id	Author	Year	Title	Country	Study design	Sample size	Population characteristics (age, setting)	Intervention details	Outcome measures	Major findings	Risk of bias
1	A. A. Alzaher, S. S. Almudarra, M. H. Mustafa and I. M. Gosadi	2018	The importance of hand hygiene education on primary schoolgirls' absence due to upper respiratory infections in Saudi Arabia. A cluster randomised controlled trial	Saudi Arabia	Cluster RCT	492 (CG 262, EG 234)	6–12 years attending 4 public primary girls' schools	Soap and water plus a 1‐h handwashing workshop, puzzle games and posters	Incidence rate, percentage of absence days, and absence rate were calculated for total and URI absences	The Incidence rate and percentage of absence days of URI absences were also lower in the schools in the EG during the study duration Schoolgirls in the EG had a 40% risk reduction in the absence and loss of school days	Some concerns
2	D. Arikan, N. Gurarslan Bas, F. Kurudirek, A. Bastopcu and H. Uslu	2018	The effect of therapeutic clowning on handwashing technique and microbial colonisation in preschool children	Turkey	Cluster RCT	195 (CG 105, EG 90)	4–6 years conducted in 2 kindergartens	Soap and water, plus a video and a clown demonstrated hygienic handwashing techniques	Bacterial colonisation load on a child's hand	At the end of the intervention, the colony count increased in 65.7% of the control group subjects; this percentage was twice that in the experimental group (31.1%)	Some concerns
3	E. Azor‐Martinez, E. Cobos‐Carrascosa, F. Gimenez‐Sanchez, J. M. Martinez‐Lopez, P. Garrido‐Fernandez et al.	2014	Effectiveness of a multifactorial handwashing program to reduce school absenteeism due to acute gastroenteritis	Spain	Cluster RCT	1616 (828 CG, 788 EG)	4–12 years of age attending 5 state schools	Soap and water were followed using hand sanitiser, while the control group (C) practised usual handwashing plus a 2‐h workshop, fortnightly education sessions, and posters	School absenteeism	EG had a 36% lower risk of absenteeism due to acute gastroenteritis and a decrease in se in absenteeism of 0.13 episodes/child/academic year absenteeism of 0.13 episodes/child/academic year	Some concerns
4	E. Azor‐Martinez, E. Cobos‐Carrascosa, M. L. Seijas‐Vazquez, C. Fernandez‐Sanchez, J. M. Strizzi et al.	2016	Hand hygiene program decreases school absenteeism due to upper respiratory infections	Spain	Cluster RCT	1616 (828 CG, 788 EG)	4–12 years of age attending 5 state schools	Soap and water were followed by the use of the hand sanitiser, while the control group (C) practised usual handwashing plus a 2‐h workshop, fortnightly education sessions and posters	School absenteeism	EG had a 38% lower risk of absenteeism due to URIs and a decrease in absenteeism of 0.45 episodes/child/academic year	Some concerns
5	E. Azor‐Martinez, Y. Gonzalez‐Jimenez, M. L. Seijas‐Vazquez, E. Cobos‐Carrascosa, J. Santisteban‐Martinez et al.	2014	The impact of common infections on school absenteeism during an academic year	Spain	Cluster RCT	1616 (CG 828, EG 788)	4–12 years old at 5 state schools	Hand sanitiser and the control group (CG) followed the usual handwashing procedure	Upper respiratory infections and gastrointestinal infections incidences	Infection‐related absenteeism (URI and GI) rates were significantly lower in the intervention groups during the school year A decrease in absenteeism of 0.7 episodes/100 children/day	High
6	D. Biswas, M. Ahmed, K. Roguski, P. K. Ghosh, S. Parveen et al.	2019	Effectiveness of a behaviour change intervention with hand sanitizer use and respiratory hygiene in reducing laboratory‐confirmed influenza among schoolchildren in Bangladesh: a cluster randomised controlled trial	Bangladesh	Cluster RCT	10 855 (CG 5778, EG 5077)	5–10 years in primary school	Hand sanitiser and respiratory hygiene education vs. no intervention	Influenza‐like illness (ILI) and laboratory‐confirmed influenza presentations	The incidence of ILI per 1000 students‐weeks was 18% lower, and laboratory‐confirmed influenza was 53% lower in the intervention group than in the control group	Some concerns
7	L. Borch, K. Thorsteinsson, T. C. Warner, C. S. Mikkelsen, P. Bjerring et al.	2020	COVID‐19 reopening causes a high risk of irritant contact dermatitis in children	Denmark	Cross‐sectional	6273	0–12 years in preschool and schoolchildren	Soap and water vs. alcohol‐based hand sanitiser	Irritant contact dermatitis	In children without any prior symptoms of ICD, as many as 42.4% developed ICD (dry, red and itchy skin) due to increased hand hygiene after the COVID‐19 reopening Furthermore, schoolchildren (6–12 years) had a relative risk of 1.5 for developing ICD when compared with preschool children (0–5 years). Hand sanitiser use was far less likely to cause ICD. Only if a child used hand sanitiser > 7 times/day, this led to an increased relative risk = 1.2 times	Some concerns
8	R. Canete, Y. Campos, R. Valdes and P. Rodriguez	2017	Prevalence and factors associated with intestinal parasitic infection among schoolchildren from Jaguey Grande municipality in Matanzas Province, Cuba	Cuba	Cross‐sectional	107	8–9‐year‐olds in primary school	Washing hands before eating and after defecation	Intestinal parasitic infections	Univariate analysis identified three factors associated with intestinal parasitic infections which include not washing hands before eating (RR = 2.53, 95% CI 1.22, 5.26), not washing hands after defaecation (RR = 2.17, 95% CI 1.23, 3.81), and drinking unboiled water (RR = 4.15, 95% CI 2.46, 7.00)	Low‐some concerns
9	B. A. Caruso, M. C. Freeman, J. V. Garn, R. Dreibelbis, S. Saboori et al.	2014	Assessing the impact of a school‐based latrine cleaning and handwashing program on pupil absence in Nyanza Province, Kenya: a cluster‐randomised trial	Kenya	Cluster RCT	17 564 (CG 5302, EG‐15490 and EG‐26772)	Grade 1–8 of school	EG‐1 handwashing, EG‐2 handwashing and latrine cleaning	Student absences	Neither intervention had a measurable impact on student absence when data were stratified by sex or grade group	Some concerns
10	Y. E. Cha, Y. Z. Fu and W. Yao	2021	Knowledge, practice of personal hygiene, school sanitation, and risk factors of contracting diarrhoea among rural students from five western provinces in China	China	Cross‐sectional	2330	9–10‐year‐old, 4th grade students	Knowledge of diarrhoea risk factors, hygiene practices, toilet facilities and quality	Diarrhoea occurrence in the previous 3 months	50.28% of those who ‘did not wash hands before every meal’ and 49.72% of those in ‘schools without hand washing facilities’ experienced diarrhoea	Some concerns
11	A. M. Denbak, A. Andersen, C. T. Bonnesen, B. Laursen, A. K. Ersboll et al.	2018	Effect evaluation of a randomised trial to reduce infectious illness and illness‐related absenteeism among schoolchildren: the hi five study	Denmark	Cluster RCT	8438 (CG 2671, EG‐12427 and EG‐22427)	6–14 years old from 43 schools	The hand hygiene curricular component consisted of 5 to 6 lessons and mandatory daily handwashing before lunch for both EG1 and EG2, and additional toilet cleaning for EG2	Infectious illness days, infectious illness episodes and illness‐related absenteeism last week	Found no significant difference in infectious illness among schoolchildren randomised to intervention arm I or II versus control schools	Some concerns
12	W. Elmonir, H. Elaadli, A. Amer, H. El‐Sharkawy, M. Bessat et al.	2021	Prevalence of intestinal parasitic infections and their associated risk factors among preschool and school children in Egypt	Egypt	Cross‐sectional	996	1–15 years in preschool and school	Washing hands after toilet, after soil contact and before eating	Intestinal parasitic infections of protozoa or helminths	The risk ratio (RR) is approximately 0.85, meaning that the risk of testing positive for intestinal parasites is 15% lower in the handwashing cohort compared to the no‐hand‐washing cohort	Some concerns
13	A. Endo, M. Uchida, N. Hayashi, Y. Liu, K. E. Atkins et al.	2021	Within and between classroom transmission patterns of seasonal influenza among primary school students in Matsumoto city, Japan	Japan	Cross‐sectional	10 923	5–12 years in primary school	Handwashing, mask wearing and vaccine	Influenza incidence	Increased susceptibility linked to handwashing (1.54; CI 1.36–1.75), inconsistent with existing findings, despite adjusting for exposure to minimise confounding. The Matsumoto dataset similarly reported a higher odds ratio (1.4; CI 1.27–1.64), possibly due to students congregating in communal handwashing areas at school	Some concerns
14	M. C. Freeman, T. Clasen, R. Dreibelbis, S. Saboori, L. E. Greene et al.	2014	The impact of a school‐based water supply and treatment, hygiene, and sanitation programme on pupil diarrhoea: A cluster‐randomised trial	Kenya	Cluster RCT	40 320 (CG 12330, EG‐114580 and EG‐213410)	4–8 years old in grades 4–8 at 135 schools	EG‐1 hygiene promotion and water treatment (HP&WT), including teacher training, handwashing facilities, and a year‐long supply of Water Guard; EG‐2 HP&WT with added school latrines (HP&WT + Sanitation); and CG receiving the intervention after data collection	1‐week period prevalence of diarrhoea and duration of diarrhoea episodes for the week	A 56% difference in the risk of diarrhoea for pupils attending intervention vs. control schools [adjusted risk ratio (aRR) 0–34, 95% CI 0.17–0.64]	Some concerns
15	S. M. Hantoosh	2023	Hand hygiene and water quality assessment in schools of Muthanna province, Southern Iraq	Iraq	Observational	1620	162 facilities (91 elementary, 42 intermediate and 29 preparatory schools)	Hand washing	*Escherichia coli* load on students' hands before and during school	Among male students in the experimental group, hand contamination decreased significantly from 31% to 25.4% (difference: 5.6%, 95% CI 1–11.1, *p* = 0.048). Female students showed a greater reduction, with contamination dropping from 23.3% to 8.6% (difference: 14.7%, 95% CI 8.4–21, *p* < 0.001) Hand hygiene levels dropped by 2.5‐fold within a few hours after school entry, compared to early‐morning levels (before school entry)	Low‐some concerns
16	E. Kavitha, R. Srikumar, G. Muthu and T. Sathyapriya	2019	Bacteriological profile and perception on hand hygiene in school‐going children	India	Cross‐sectional	133	9–12 age, in grades 6–9 at school	Frequency of handwashing after toileting or before a meal	Pathogenic bacteria load on students' hands	Although frequent handwashing was observed among the schoolchildren, they hardly used soap for washing their hands The nonavailability of soap at the washing station and in the toilets was the reason for the contaminated hands among the schoolchildren	Some concerns
17	A. A. Kim and T. Wu	2015	Assessment of diarrheal rates in a population of children in the Indian Himalayas: a student initiative	India	Cross‐sectional	258	6–18 years old	Hand washing as a predictive factor	Episodes of diarrhoea within the past 14 days	Handwashing before meals and toileting decreased diarrheal disease risk by 78.3% but are not predictive for dysentery	Some concerns
18	K. Klar, D. Knaack, S. Kampmeier, A. K. Hein, D. Gorlich et al.	2022	Knowledge about hand hygiene and related infectious disease awareness among primary school children in Germany	Germany	Cross‐sectional	489	8–11, in a third‐grade school	A questionnaire about hand hygiene	Frequency of visits to the sanitary facilities and hygiene practices at the school	Slightly less than half of school children (48.0%) reported washing their hands at school before eating, with 23.7% doing so always and 24.3% often Despite being taught this habit in daycare, about half fail to practice it regularly at school	Low‐some concerns
19	T. H. Koep, S. Jenkins, M. H. Me, C. Clemens, E. Fracica et al.	2016	Promotion of influenza prevention beliefs and behaviours through primary school science education	USA	Non‐randomised interventional cohort study	95 (CG 80, EG 45)	8–10‐year‐old in 3rd and 4th grade	4–6 week influenza prevention prescription education, in addition to automated soap and sanitiser dispensers equipped with sensors logging every individual HH event to measure changes pre‐/post curricula	Understanding of influenza prevention related to hand washing and vaccination, and hand hygiene frequency pre‐/post intervention	Talking drawings showed improved understanding of influenza prevention for handwashing (43%–77%) and vaccination (5%–38%) Surveys of 566 students (*n* = 1024) revealed strong baseline hygiene attitudes, while automated monitoring estimated median soap compliance at 63% and hand sanitiser use (0.04–0.24 uses per student per day) but found no significant changes pre‐ and post‐IPPE	High
20	M. A. Mahmud, M. Spigt, A. M. Bezabih, G. J. Dinant and R. B. Velasco	2020	Associations between intestinal parasitic infections, anaemia, and diarrhoea among school aged children, and the impact of handwashing and nail clipping	Ethiopia	Cluster RCT	367 (CG 87, EG‐1 91, EG‐2 95 and EG‐3 94)	6–15 years	EG‐1 handwashing, EG‐2 nail clipping, EG‐3 handwashing and nail clipping	Reinfection with intestinal parasitic infections	Both handwashing with soap (aOR 0.32, 95% CI 0.20–0.62, *p* = 0.001) and weekly fingernail clipping (AOR 0.51, 95% CI 0.27–0.95, *p* = 0.035) interventions were reported to have a significant impact in reducing intestinal parasite reinfection rates among the study participants	Low
21	A. Matsuda, K. Asayama, T. Obara, N. Yagi and T. Ohkubo	2023	Behavioural changes of preventive activities of influenza among children in satellite cities of a metropolitan area of Tokyo, Japan, by the COVID‐19 pandemic	Japan	Cross‐sectional	13 206	< 15 years old at preschool, elementary school, and junior high school	Hand washing, face mask wearing and vaccination	Influenza infection prevalence	Hand washing alone was associated with a greater risk of influenza infection	Some concerns
22	J. A. Nicholson, M. Naeeni, M. Hoptroff, J. R. Matheson, A. J. Roberts et al.	2014	An investigation of the effects of a hand washing intervention on health outcomes and school absence using a randomised trial in Indian urban communities	India	Cluster RCT	1680 (CG 833, EG 847)	5–7 years old at school	41‐week education programme + provision of free soap	Diarrhoea and acute respiratory infections (ARIs), school absences and soap consumption for 41 weeks	Intervention group experienced fewer episodes of diarrhoea (−25%), ARIs (−15%), illness‐related school absences (−27%) and eye infections (−46%), with similar reductions observed in diarrhoea (−31%) and ARIs (−14%) for whole families Confidence intervals for all reductions were statistically significant	Some concerns
23	P. P. Or, P. T. Ching and J. W. Chung	2020	Can flu‐like absenteeism in kindergartens be reduced through hand hygiene training for both parents and their kindergarteners?	China	Quasi‐experimental study	60	5–6‐year‐old in kindergarten	4 sessions of hand hygiene education lasting 45 min	Recorded signs and symptoms of flu‐like illnesses and absences from kindergarten	Kindergarteners' absence rates in all participating kindergartens owing to flu decreased from 21.5% to 12% over the study period in 3 months	Some concerns
24	P. P. L. Or, P. T. Y. Ching and J. W. Y. Chung	2019	A program to improve the hand hygiene compliance of Hong Kong preschoolers with an insight into their absenteeism	China	Quasi‐experimental study	110	5–6 years at preschool	4 training sessions on hand hygiene	Hand hygiene knowledge and their handwashing skills	Significant improvements were observed in hand hygiene knowledge, performance (*P* < 0.05), and influenza‐related absence rates, which decreased before (31%), during (30%), and after (25%) the programme	Some concerns
25	Y. Otsuka, L. Agestika, H. Harada, L. Sriwuryandari, N. Sintawardani et al.	2019	Comprehensive assessment of handwashing and faecal contamination among elementary school children in an urban slum of Indonesia	Indonesia	Cross‐sectional	169	Grade 2, 4 and 6	handwashing observation using a checklist, hand bacteria sampling and questionnaires	Faecal *Escherichia coli* contamination load on the hands	Proper handwashing techniques with soap at appropriate times can reduce faecal contamination, but these practices were poor in lower grades, with boys at higher risk Grade‐ and gender‐based handwashing education in elementary schools is essential	Low‐some concerns
26	S. K. Padaruth and S. D. Biranjia‐Hurdoyal	2015	Hygiene practices and faecal contamination of the hands of children attending primary school in Mauritius	Mauritius	Observational	200 (6–8 years, 131 and 9–10 years, 69)	6–10 years old school children	The study examined children's handwashing habits and hygienic practices before and after eating and sneezing, as well as oral‐digital habits like finger‐sucking and nail‐biting	Bacterial growth on the hand from the swab	Children aged 9–10 were more likely to wash hands before eating (OR = 2.0; *p* < 0.05), while those aged 6–8 had higher rates of oral‐digital habits (OR = 2.5; *p* < 0.05) and less frequent post‐toilet handwashing Washing with water only was associated with higher bacterial growth (OR = 5.9; *p* < 0.05), especially among younger children	Some concerns
27	P. Priest, J. E. McKenzie, R. Audas, M. Poore, C. Brunton et al.	2014	Hand sanitiser provision for reducing illness absences in primary school children: a cluster randomised trial	New Zealand	Cluster RCT	2443 (EG 1301, CG 1142)	5–11 years at school	EG had alcohol‐based hand sanitiser dispensers in classrooms for the winter school terms, and both EG and CG received an in‐class hand hygiene education session.	Absence episodes due to respiratory or gastrointestinal illness	The provision of an alcohol‐based hand sanitiser dispenser in classrooms was not effective in reducing rates of absence episodes due to respiratory or gastrointestinal illness, the length of illness or illness absence episodes Incidence rate ratio 1.06, 95% CI 0.94–1.18	Low
28	K. Riiser, S. Helseth, K. Haraldstad, A. Torbjørnsen and K. R. Richardsen	2020	Adolescents' health literacy, health protective measures, and health‐related quality of life during the Covid‐19 pandemic	Norway	Cross‐sectional	2205	16–19 years old	Participants reported on their health information sources, health literacy, handwashing knowledge and behaviour, number of social interactions, and health‐related quality of life	Knowledge and adherence to the health authorities' guidelines	Health literacy and handwashing knowledge and health literacy and handwashing behaviour were significantly associated	Some concerns
29	G. B. Roro, F. Eriso, A. M. Al‐Hazimi, M. Kuddus, S. C. Singh et al.	2022	Prevalence and associated risk factors of *Entamoeba histolytica* infection among school children from three primary schools in Arsi Town, West Zone, Ethiopia	Ethiopia	Cross‐sectional	334	5–17 at 3 elementary schools	Hand wash before eating, hand wash after defecation and awareness about amoebiasis	Prevalence of *E. histolytica* in school children	Multivariate analysis revealed significant associations between *E. histolytica* infection and handwashing before eating (AOR = 0.32) and after defecation (AOR = 0.396), and awareness of amoebiasis (AOR = 0.35) These findings highlight the importance of hygiene practices and education in reducing infection risk	Low‐some concerns
30	N. Torner, N. Soldevila, J. J. Garcia, C. Launes, P. Godoy et al.	2015	Effectiveness of non‐pharmaceutical measures in preventing paediatric influenza: a case–control study	Spain	Case–control	239	0–17 at preschool and school	Frequency of hand washing, alcohol‐based hand sanitiser use and hand washing after touching contaminated surfaces. During the 7 days before the onset of symptoms	Influenza infection rates	Washing hands more than five times daily was a significant protective factor against influenza (aOR = 0.62; *p* = 0.04) When considering two age groups (preschool age 0–4 years and school age 5–17 years), only the school age group who had frequent handwashing (aOR = 0.47) and washing after touching contaminated surfaces (aOR = 0.19) were associated with reduced infection risk	Some concerns
31	V. Trinies, A. Chard, H. Chang and M. Freeman	2014	Impact of a school‐based water, sanitation and hygiene program on diarrhoea, respiratory infections and absenteeism: A longitudinal evaluation	Mali	Longitudinal	9730 (CG 4823, EG 4907)	8 to 13 years from grades 3 to 6	Installing or rehabilitating water points and latrines, distributing WASH supplies, and promoting hygiene through teacher training, school management engagement, and hygiene club initiatives	Pupil absence, secondary outcomes of self‐reported absence, diarrhoea, and respiratory infection symptoms in the past week	The odds of pupils reporting being absent due to diarrhoea (OR: 0.73, 95% CI: 0.56, 0.94) or having had diarrhoea (OR: 0.71, 95% CI: 0.60, 0.85) or respiratory infection symptoms (OR: 0.75, 95% CI: 0.65, 0.86) in the past week were lower in beneficiary schools compared with comparison schools	Some concerns
32	A. Tunio, J. Ahmed, M. Z. Shaikh, N. Channa, S. Hussain et al.	2024	Impact of hand hygiene interventions on handwashing practices and microbial risk: A study in an orphanage‐based school in Pakistan	Pakistan	Cluster RCT	48 (CG 36, EG 36)	School age in an orphanage	The intervention group participated in activities promoting hand hygiene, including awareness sessions, videos, illustrations, posters, kits and promotional materials, while the control group only attended awareness sessions	Bacterial loads on children's hands before and after hygiene interventions	The intervention led to an 80% bacterial reduction and a 100% decrease in illness probability from *E. coli* , while the control group showed a 55% bacterial reduction and 78% illness reduction Higher bacterial ingestion rates were linked to hand contamination and hand‐to‐mouth transfer	High
33	H. Vally, C. McMichael, C. Doherty, X. Li, G. Guevarra et al.	2019	The impact of a school‐based water, sanitation and hygiene intervention on knowledge, practices and diarrhoea rates in the Philippines	Philippines	Quasi‐experimental study	2001 (CG 1309, EG 692)	5–12 years old in kindergarten to grade 6	Four schools had recently completed the PRC WASH intervention, while the comparison schools had not yet received the intervention but were slated to participate later	Absences due to diarrhoea, and handwashing frequency at critical times	Students in intervention schools had significantly lower self‐reported school absences due to diarrhoea, with a seven‐fold risk reduction (1.4% vs. 9.4%; RR = 0.15; *p* < 0.001) Multilevel analyses confirmed 10‐fold lower odds of absence in intervention versus comparison schools (OR = 0.10; *p* < 0.001)	Low‐some concerns

### 3.4 Data Extraction

Data extraction was done following the Cochrane guidelines [11]. For each included study, data was extracted and tabulated into a standardised electronic form, including author, year of publication, title, country, study design, sample size, population characteristics (e.g., age, setting), intervention details (e.g., type of hand hygiene, frequency), outcome measures (e.g., infectious disease incidence, absenteeism) and comment on the major findings. Extracted data was cross‐checked by the reviewers and addressed if missing data was identified. The results of this process are displayed in Table 1 and Appendix S3.

## 4 Results

### 4.1 Search Results

**FIGURE 1:**
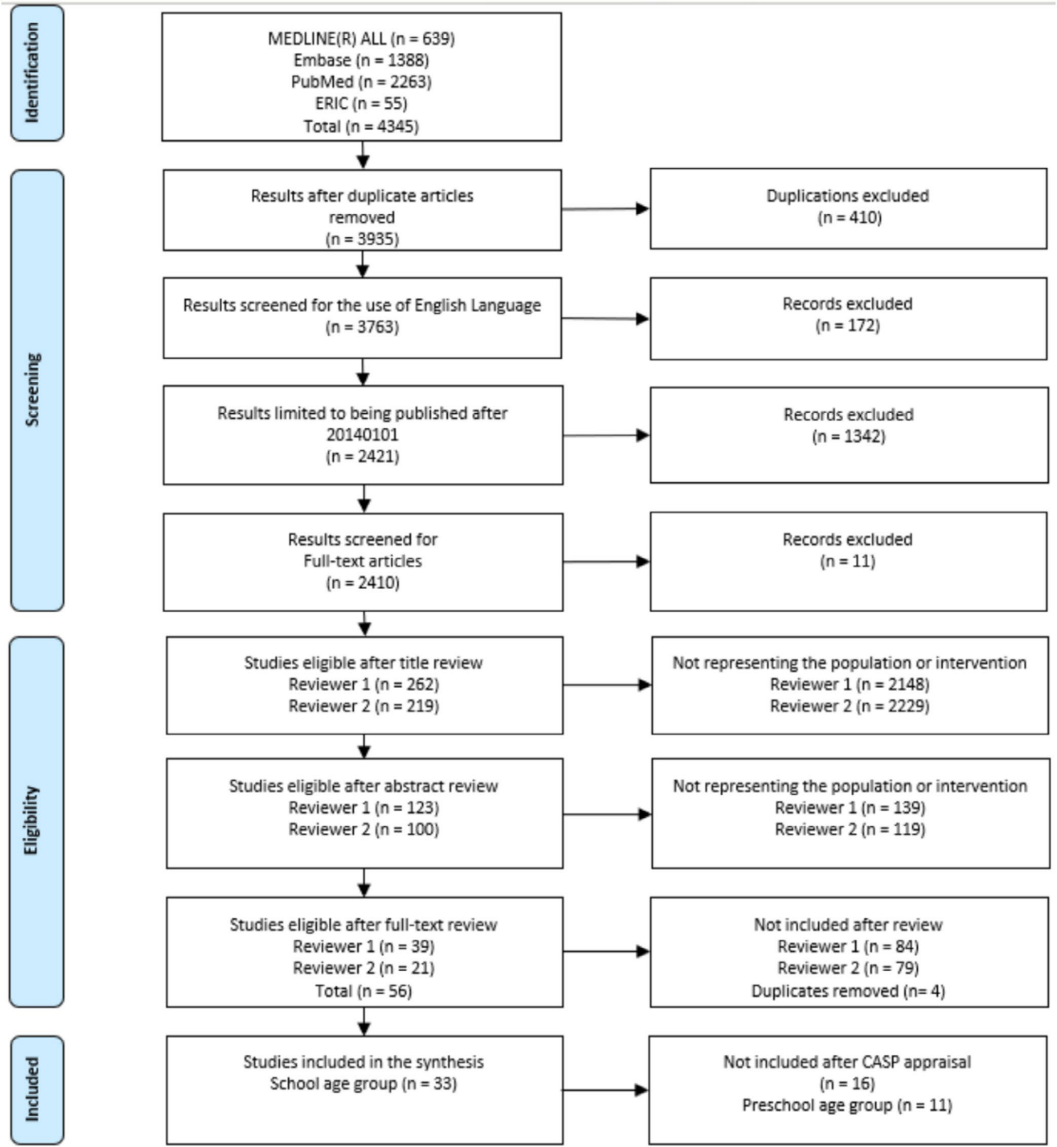
PRISMA diagram.

Database searches identified a total of 4345 articles from Medline (*n* = 639), Embase (*n* = 1388), PubMed (*n* = 2263) and ERIC (*n* = 55). After de‐duplication, a total of 3935 articles were screened for the use of the English language, published since January 2014, and being full‐text articles, identifying 2410 unique records. During the title and abstract screening, Reviewer 1 (AS) excluded 2287 articles, while Reviewer 2 (NS) excluded 2348 articles. Subsequently, a full‐text review was undertaken on 100 and 123 records, respectively, of which 56 studies were found to fulfil the eligibility criteria. Following the CASP appraisal (Appendix S2), 33 studies were included in the final synthesis (Figure 1).

**FIGURE 2:**
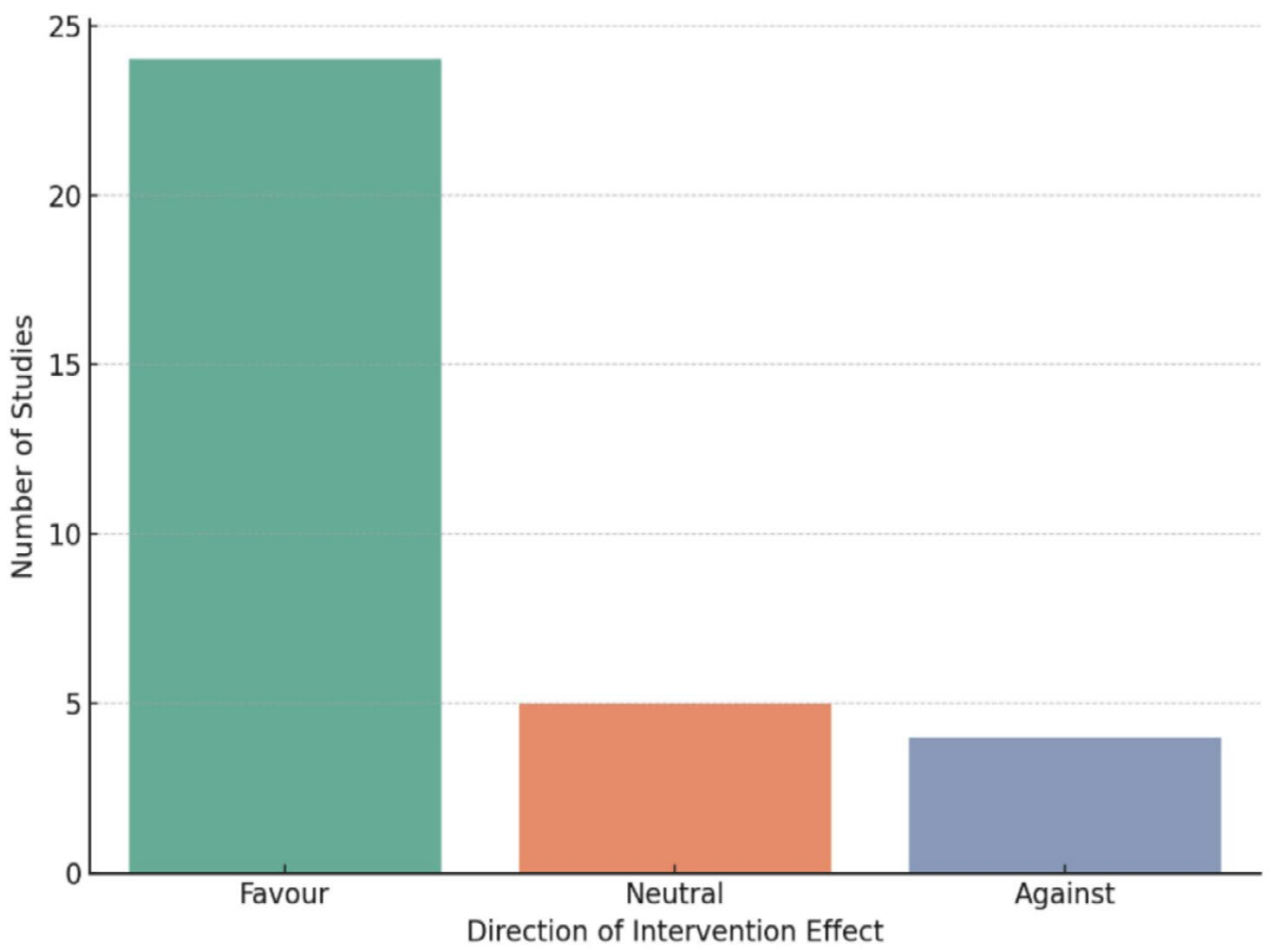
Impact of hand hygiene interventions in school studies.

Figure 2 displays the number of studies in favour, neutral and against hand hygiene interventions.

- Favour (*n* = 23): Most studies showed a positive impact, such as reduced absenteeism, fewer infections or improved hygiene knowledge/behaviour.
- Neutral/mixed (*n* = 6): Some studies had limited, mixed or conditional effects often dependent on factors like adherence, infrastructure or additional educational support.
- Against (*n* = 4): A few studies showed either no benefit or unintended harms (e.g., irritant contact dermatitis [ICD] or increased infection risk due to poor technique or implementation).

Due to the high heterogeneity of the included studies and the variability in outcome measures, a meaningful synthesis of the results was not feasible.

**FIGURE 3:**
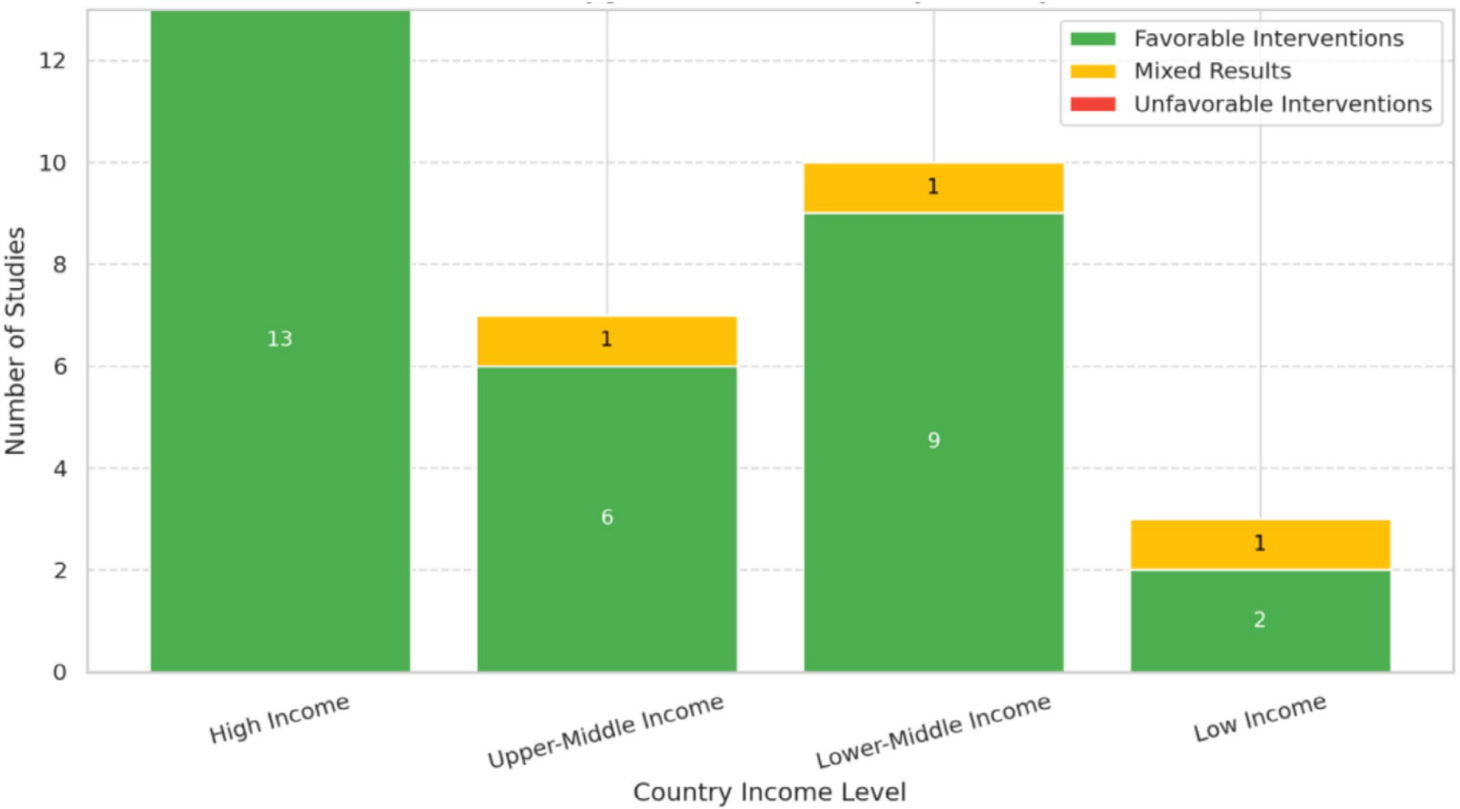
Outcomes of hand hygiene interventions by country income level.

Figure 3 displays the distribution of study outcomes between favourable, mixed and unfavourable results according to World Bank income levels.

- High‐income countries (*n* = 13): All studies showed favourable results, supported by strong infrastructure, resources (soap, sanitisers) and school engagement.
- Upper‐middle‐income countries (*n* = 7): Six favourable outcomes; one mixed result linked to inconsistent implementation and limited resource support.
- Lower‐middle‐income countries (*n* = 10): Nine favourable, one mixed; programmes effective with education and basic supplies despite structural challenges.
- Low‐income countries (*n* = 3): Two favourable, one mixed; outcomes heavily influenced by water access, sanitation and programme quality; sustainability affected by infrastructure gaps.

Most of the studies showed positive results, whereas a few showed significant implementation barriers such as dermatitis from excessive washing, supply shortages and knowledge‐practice discrepancies. There were no unfavourable interventions.

**FIGURE 4:**
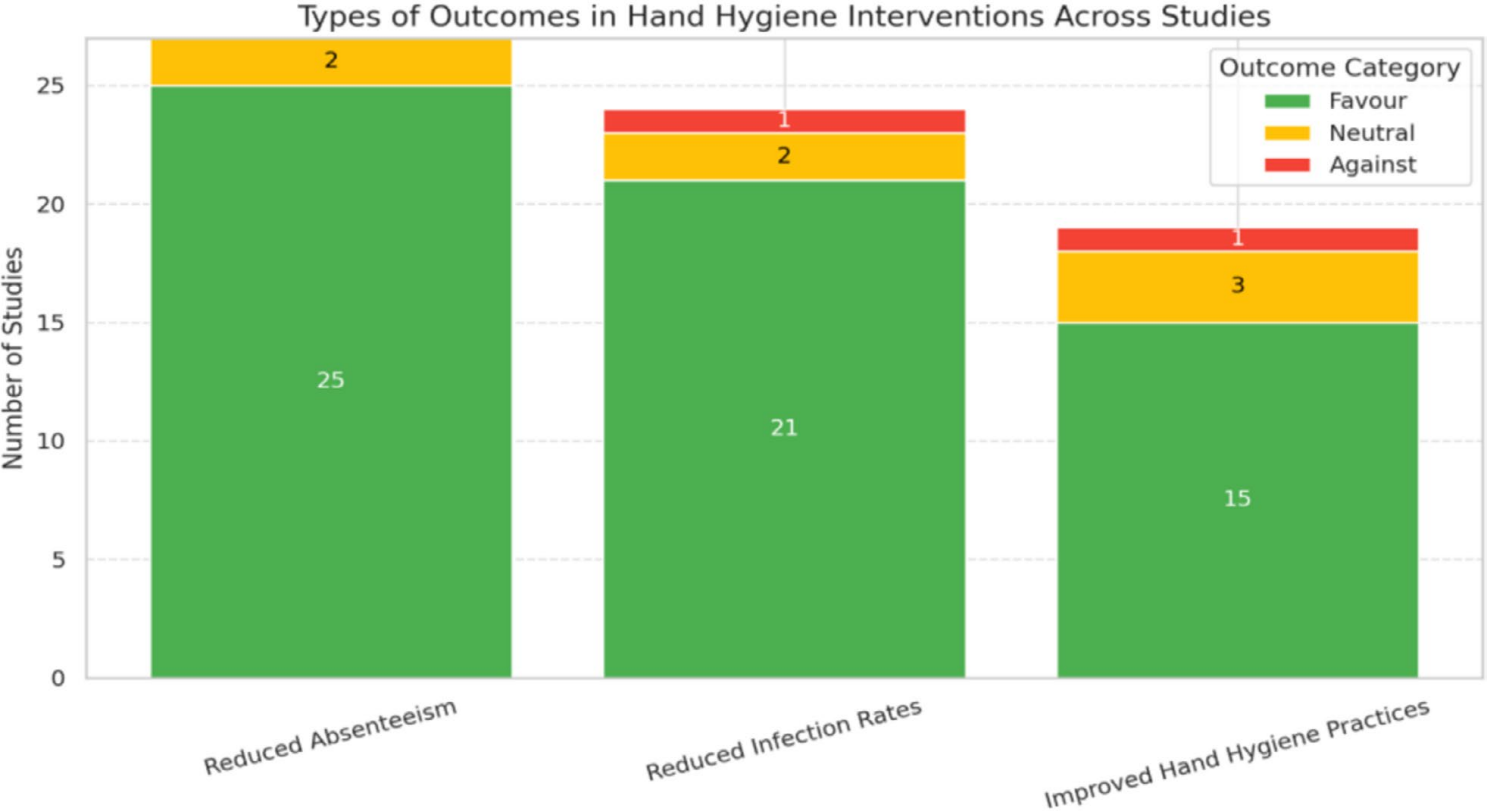
Types of outcomes in hand hygiene interventions across studies.

Figure 4 shows how outcomes from school‐based hand hygiene interventions are distributed across three key categories: absenteeism, infection rates and hand hygiene practices, with each outcome further classified as favourable, neutral or against.

- Reduced absenteeism: 25 studies reported significant decreases in school absences following hand hygiene interventions; 2 studies showed neutral or inconclusive effects.
- Reduced infection rates: 21 studies demonstrated lower respiratory or gastrointestinal infection rates; 2 studies were neutral, and 1 reported no benefit, likely due to implementation or resource constraints.
- Improved hand hygiene practices: 15 studies observed better handwashing frequency, technique and compliance; 3 studies were neutral, often due to short follow‐up or minimal behaviour change, and 1 reported negative outcomes linked to dermatitis or poor adherence.

Overall, this supports the conclusion that school‐based hand hygiene programmes are highly effective, particularly when implemented with proper support, infrastructure and reinforcement strategies.

### 4.2 Study Designs

Overall, 33 studies were focused on school children. Study designs included 13 cluster randomised controlled trials, 12 cross‐sectional studies, 3 Quasi‐experimental studies, 2 observational studies, a non‐randomised interventional cohort study, a case–control study and a longitudinal study.

### 4.3 Settings

The studies were carried out across a diverse range of countries, Saudi Arabia, Spain, Turkey, Bangladesh, Denmark, Cuba, Kenya, China, Egypt, Japan, Iraq, India, Germany, the United States, Ethiopia, Indonesia, Mauritius, New Zealand, Norway, Mali, Pakistan and the Philippines. 13 (40%) of the included studies were carried out in high‐income economy countries, 7 (22%) in upper‐middle‐income countries, 10 (31%) in lower‐middle‐income economies and the remaining 3 (7%) studies were conducted in low‐income economies, as classified by the World Bank [12, 13].

### 4.4 Intervention and Outcomes

The implementation of hygiene interventions in schools and childcare settings effectively decreased illness‐related absenteeism and improved child health outcomes [14, 15, 16, 17, 18]. WASH (water, sanitation and hygiene) initiatives integrating multiple components also yielded positive outcomes, reducing diarrhoeal and respiratory absences across diverse settings, with girls often benefiting most [19, 20, 21, 22].

School curriculum‐based education and parental engagement showed sustained benefits for hygiene knowledge, handwashing practices and absenteeism [23, 24, 25]. Handwashing combined with other strategies, such as mask use, nail clipping or structured protocols, further reduced infection‐related absences and reinfection rates [26, 27, 28]. Sanitiser provision alone showed mixed results but was generally more effective when paired with education and consistent reinforcement [29].

Overall, multifaceted interventions combining education, parental involvement, facility upgrades and behavioural reinforcement were the most successful in improving hand hygiene and reducing school absenteeism.

### 4.5 Risk of Bias Assessment

Overall, the risk of bias varied across the included studies. Approximately half of the studies demonstrated low risk, particularly cluster randomised controlled trials with robust allocation, follow‐up and outcome measurement procedures. Several studies exhibited moderate risk, often due to small sample sizes, reliance on self‐reported outcomes or limited blinding, while a smaller number were assessed as high risk, typically reflecting non‐randomised designs with limited control for confounding. These variations were considered when interpreting the findings to ensure conclusions were drawn cautiously and appropriately.

Considering study design, risk‐of‐bias assessments, and the consistency of outcomes across settings, the overall certainty of the evidence can be rated as moderate. Many controlled studies support the effectiveness of school‐based hand hygiene interventions; however, heterogeneity in intervention approaches, reliance on self‐reported outcomes and variable follow‐up durations temper confidence.

Details of the quality assessment for individual studies are presented in Appendix S2.

## 5 Discussion

### 5.1 Impact of Hand Hygiene Interventions on Absenteeism and Infectious Disease Rates

This systematic review of 33 studies across diverse geographical and economic settings provides consistent evidence that school‐based hand hygiene interventions significantly reduce absenteeism and the incidence of respiratory, gastrointestinal and parasitic infections among children. Multifaceted interventions combining hygiene education with access to soap or alcohol‐based sanitisers and behavioural reinforcement were particularly effective.

In Spain, Azor‐Martínez et al. reported that such a programme led to a 36% reduction in acute gastroenteritis‐related absenteeism and a 24% decline in URI‐related absences [16, 17]. Similarly, Indian studies observed a 23%–24% reduction in both total and respiratory‐related absenteeism following hygiene education [18]. Structured WASH interventions in Pakistan and the Philippines produced similar outcomes [19, 20], while in Kenya and Mali, integrating hygiene messaging with infrastructural improvements significantly decreased absenteeism [21, 22].

### 5.2 Effectiveness of Educational and Family‐Centred Approaches

Educational programmes alone also proved beneficial. In India, a handwashing intervention led to significant reductions in respiratory‐related school absences [23]. In Hong Kong, involving both parents and children in hygiene training reduced flu‐like absenteeism from 21.5% to 12% [24], and visual aids like fluorescent gels further improved handwashing technique and attendance [25]. Koep et al. found that embedding hygiene promotion into science curricula led to sustained behavioural improvements over 3 months [26].

### 5.3 Impact on Intestinal Parasitic Infections (IPIs)

IPIs remain a serious concern in resource‐limited settings. Studies in Cuba, Egypt and Ethiopia found prevalence rates exceeding 50%, attributed to poor hygiene practices around meals and defecation [27, 28, 29]. Mahmud et al. demonstrated that a combination of handwashing with soap and regular nail clipping significantly reduced reinfection rates [30]. In Himalayan schools, hygiene promotion led to a 78.3% reduction in diarrheal illnesses [31].

### 5.4 Role of Educators and Age‐Appropriate Strategies

Teacher‐led programmes have shown measurable success. In Kenya, Denbæk et al. observed a decline in respiratory and diarrheal illness‐related absenteeism through structured reinforcement [32]. Arıkan et al. reported that interactive and visually engaging hygiene demonstrations reduced microbial colonisation by 50% among schoolers [15]. These findings support tailoring interventions to developmental stages for maximal impact.

### 5.5 Implementation Challenges and Unintended Consequences

Despite overall success, some challenges emerged. Frequent handwashing was linked to ICD, with Borch et al. documenting a 42.4% prevalence, particularly when children washed hands more than 7–10 times daily [33]. This suggests the need to balance soap use with sanitisers or moisturisers. In some low‐resource schools, poor infrastructure hindered outcomes. Hantoosh et al. found *E. coli* in 12% of treated school water, and hygiene adherence declined during the school day due to limited facilities [34].

Priest et al. emphasised that sanitiser‐only interventions showed mixed outcomes, with success depending on strict implementation and adherence [35]. Freeman and Otsuka highlighted that schools lacking adequate water infrastructure struggled to maintain hygiene gains, though improvements in water access reduced absenteeism by up to 50% [36, 37].

### 5.6 Behavioural Gaps and Resource Constraints

Padaruth et al. and Kavitha et al. found extensive bacterial contamination (e.g., *E. coli* , *S. aureus* ) on children's hands due to soap shortages despite hygiene education [38, 39]. Klar et al. and Cha et al. identified disconnects between knowledge and practice, often attributed to lack of time, soap or role models [40, 41]. These behavioural barriers limit the effectiveness of otherwise well‐designed programmes.

Despite generally positive outcomes, some interventions showed limited or neutral effects even in well‐resourced settings, often due to inconsistent adherence by students or staff, insufficient reinforcement of hygiene behaviours, low engagement with educational components and lapses in implementation fidelity, underscoring that even well‐designed programmes require sustained behavioural support and supervision to achieve intended outcomes [35, 36, 39, 40, 41].

### 5.7 Population‐Specific Insights: Age, Gender and School Environment

Azor‐Martínez et al. identified children under five as particularly vulnerable to URIs, warranting prioritisation in intervention strategies [42]. Endo et al. found that two‐thirds of influenza cases originated within classrooms, supporting targeted in‐class hygiene interventions [43]. Matsuda et al. showed that combining mask use with handwashing offered greater protection against influenza outbreaks than either measure alone [44], while Torner et al. noted a 47% reduction in flu risk with frequent sanitiser use, though compliance remained variable [45].

### 5.8 Influence of Health Literacy and Socio‐Cultural Factors

Health literacy played a critical role. Riiser et al. demonstrated that adolescents with higher health literacy had better compliance with COVID‐19‐related hygiene measures, suggesting the benefits of integrating health education into school curricula [46]. Gender‐specific effects were observed by Caruso et al. and Freeman et al., who reported up to a 50% reduction in absenteeism among girls due to improved sanitation access [21, 47]. Alzaher et al. showed that hygiene education alone halved URI‐related absences among Saudi schoolgirls, highlighting the scalability of low‐cost interventions in resource‐constrained settings [48].

### 5.9 Policy and Practice Implications Across Resource Settings

From a policy and practice perspective, the findings offer clear, context‐specific guidance for both high‐ and low‐resource school settings. In high‐income contexts, where hygiene infrastructure is typically established, policy efforts should focus on sustained behavioural reinforcement, integration of hygiene education into school curricula, and mitigation of unintended harms such as ICD through balanced hand hygiene protocols. In lower‐resource settings, the evidence supports prioritising low‐cost, high‐impact strategies, including structured hygiene education, handwashing at key moments, and incremental WASH improvements, even in the absence of comprehensive infrastructure. Across all contexts, teacher engagement, parental involvement and reliable access to basic supplies emerge as critical implementation levers for reducing infectious disease transmission and illness‐related absenteeism in schools.

### 5.10 Implications for Practice, Equity and Future Research

School‐based hand hygiene interventions demonstrate consistent benefits across diverse settings, yet several considerations are critical for effective implementation and sustainability. Equity considerations are critical, as low‐resource schools often face barriers such as limited water supply, insufficient soap or sanitisers and inadequate infrastructure. Sustainable implementation may require context‐specific strategies, including resource prioritisation, teacher and parental engagement and low‐cost educational materials to maintain hygiene improvements across diverse socioeconomic settings [19, 20, 21, 22, 36, 37]. Even well‐designed programmes require consistent behavioural reinforcement and monitoring to maintain adherence and maximise effectiveness [35, 36, 39, 40, 41].

Furthermore, to mitigate against adverse effects, balanced handwashing frequency, alcohol‐based sanitiser options and moisturiser use are recommended to minimise ICD.

Unlike prior reviews, this study emphasises both behavioural and infrastructural dimensions, providing a foundation for a conceptual framework that integrates education, facility upgrades and reinforcement strategies to optimise hygiene outcomes.

Future research should examine the long‐term sustainability and cost‐effectiveness of these programmes, standardise outcome measures and evaluate strategies to maintain hygiene behaviours over time across diverse socioeconomic contexts. Addressing these considerations will support equitable, scalable and evidence‐informed school health interventions globally.

## 6 Limitations

### 6.1 Limitations of the Included Evidence

The reviewed studies demonstrated positive results, yet several key limitations exist in the primary evidence. The studies heavily depended on self‐reported data about hygiene practices and illness symptoms, which remain vulnerable to recall and reporting biases. The studies lacked objective outcome measures such as microbiological assays or validated attendance registers, which weakens the validity of their findings. The studies used different intervention approaches and outcome definitions because they implemented educational posters as well as complete WASH programmes that included latrine construction. The diverse nature of studies makes it difficult to compare results and reduces the ability to apply findings to other contexts. The lack of extended follow‐up periods in many studies creates uncertainty about how well health benefits can persist. The results might have been influenced by the fact that most studies focused on high‐income countries, which possess better infrastructure. The research included insufficient data about adolescent girls and gender‐specific outcomes, which restricted the ability to understand subgroup effects.

### 6.2 Limitations of the Review Process

The review process requires attention to several methodological limitations. The review process included thorough screening and analysis, yet it only considered studies published in English, which might have omitted important grey or non‐English literature. The process of extracting and synthesising data required human interpretation, which created potential biases from reviewers. The review did not perform a formal meta‐analysis because the studies exhibited significant differences in their designs and populations, and measurement outcomes.

## 7 Conclusion

This review of the evidence at a systematic level shows a large impact of school‐based interventions on hand hygiene and WASH on reducing the burden of communicable disease and absence from school by children aged between 5 and 18 years. In diverse geographical and socioeconomic contexts, there is evidence of a decrease in rates of diarrhoeal disease, respiratory infections, and days missed at school due to promoting correct handwashing behaviour and improving personal and communal hygiene facilities and integration of hygiene teaching into school curricula [49, 50]. These individual health gains extend to educational attainment as a wider marker of community well‐being [51].

Findings provide evidence in support of advocating hand hygiene promotion as a cost‐effective, high‐impact strategy of school health promotion [52]. Governments, teachers and public health stakeholders will need to collaborate on establishing hygiene practices through continuous funding, policy direction and intersectoral efforts under health education, behavioural and infrastructural dimensions [53]. While limitations of heterogeneity and research consistency apply at the study level, this cumulative evidence finds support for schools as key vehicles of public health intervention [54]. Standardisation of outcomes, long‐term sustainability, and cost‐effectiveness need to become research areas of emphasis to inform scalable, evidence‐informed global implementation [55].

## Author Contributions

The authors equally contributed to the conception, design, analysis, and drafting of the manuscript.

## Funding

The authors have nothing to report.

## Disclosure

The systematic review protocol was prospectively submitted to PROSPERO with registration granted on the 10th of December 2024 (CRD42024620293) [7, 56].

## Ethics Statement

The authors have nothing to report.

## Consent

The authors have nothing to report.

## Conflicts of Interest

The authors declare no conflicts of interest.

## Supporting information


**Appendix S1:** Hand hygiene practices in paediatric populations: Search strategy. This appendix provides the complete search strategy used to identify studies on hand hygiene practices and interventions in paediatric populations. It includes database names, search dates, search strings, Boolean operators and any applied filters or language restrictions.

**Appendix S2:** Hand hygiene: CASP and risk of bias (RoB) checklist. This appendix presents the results of the Critical Appraisal Skills Programme (CASP) checklist and the risk of bias assessments for all included studies. Each criterion is accompanied by an explanation of how quality judgements were reached.

**Appendix S3:** Hand hygiene: Data extraction. This appendix contains the full data extraction tables summarising study characteristics, populations, intervention details, comparator conditions, outcome measures and key findings for every included study.

## Data Availability

The data that supports the findings of this study are available in the Supporting Information of this article.

## References

[1] Z. Wang , M. Lapinski , E. Quilliam , L. A. Jaykus , and A. Fraser , “The Effect of Hand‐Hygiene Interventions on Infectious Disease‐Associated Absenteeism in Elementary Schools: A Systematic Literature Review,” American Journal of Infection Control 45, no. 6 (2017): 682–689, https://www.sciencedirect.com/science/article/pii/S019665531730041X. 28242074 10.1016/j.ajic.2017.01.018

[2] J. M. Boyce and D. Pittet , “Guideline for Hand Hygiene in Health‐Care Settings: Recommendations of the Healthcare Infection Control Practices Advisory Committee and the HICPAC/SHEA/APIC/IDSA Hand Hygiene Task Force,” MMWR Recommendations and Reports 51, no. RR‐16 (2002): 1–44, https://www.cdc.gov/mmwr/preview/mmwrhtml/rr5116a1.htm. 12418624

[3] World Health Organization , Infection Prevention and Control [Internet], ed. WHO (World Health Organization, 2025), https://www.who.int/health‐topics/infection‐prevention‐and‐control#tab=tab_1.

[4] M. Willmott , A. Nicholson , H. Busse , G. J. MacArthur , S. Brookes , and R. Campbell , “Effectiveness of Hand Hygiene Interventions in Reducing Illness Absence Among Children in Educational Settings: A Systematic Review and Meta‐Analysis,” Archives of Disease in Childhood [Internet] 101, no. 1 (2016): 42–50, https://pubmed.ncbi.nlm.nih.gov/26471110/. 26471110 10.1136/archdischild-2015-308875 PMC4717429

[5] T. R. Shope , “Infectious Diseases in Early Education and Child Care Programs,” Pediatrics in Review 35, no. 5 (2014): 182–193, 10.1542/pir.35-5-182. 24790072

[6] G. De Broucker , S. Y. Sim , L. Brenzel , M. Gross , B. Patenaude , and D. O. Constenla , “Cost of Nine Pediatric Infectious Illnesses in Low‐ and Middle‐Income Countries: A Systematic Review of Cost‐Of‐Illness Studies,” PharmacoEconomics 38, no. 10 (2020): 1071–1094, 10.1007/s40273-020-00940-4. 32748334 PMC7578143

[7] A. Silburn and N. Singh , “Hand Hygiene Practices in Paediatric Populations: Assessing Their Impact on Infectious Disease Outbreaks in Preschools and Schools [Protocol]. PROSPERO [Internet],” 2025, https://www.crd.york.ac.uk/prospero/display_record.php?ID=CRD42024620293. 10.1111/jpc.70311 PMC12976192 41664625

[8] A. Kaste , “Medline Complete,” Journal of the Medical Library Association 103, no. 1 (2015): 62–63.

[9] Thomson Reuters , EndNote X8 [Software] (Thomson Reuters, 2017).

[10] CASP , Critical Appraisal Skills Programme (CASP) [Internet] (CASP, 2025), https://casp‐uk.net/.

[11] J. P. T. Higgins , J. Thomas , J. Chandler , et al., eds., Cochrane Handbook for Systematic Reviews of Interventions [Internet], 2nd ed. (John Wiley & Sons, 2019), https://training.cochrane.org/handbook.

[12] S. R. Ismail , R. Radzi , P. S. N. Megat Kamaruddin , et al., “The Effects of School‐Based Hygiene Intervention Programme: Systematic Review and Meta‐Analysis,” PLoS One 19, no. 10 (2024): e0308390, 10.1371/journal.pone.0308390. 39378207 PMC11460677

[13] World Bank , World Bank Country and Lending Groups (World Bank, 2025), https://datahelpdesk.worldbank.org/knowledgebase/articles/906519‐world‐bank‐country‐and‐lending‐groups.

[14] A. A. Alzather , H. A. Mahmoud , and R. A. Alghamdi , “Knowledge and Practices of Hand Hygiene Among Intermediate School Students in Saudi Arabia: A Cross‐Sectional Study,” Journal of Family Medicine and Primary Care 11, no. 1 (2022): 167–172, 10.4103/jfmpc.jfmpc_1316_21.

[15] D. Arikan , N. Alpugan , and A. Kiliç , “Investigation of Personal Hygiene Habits and Knowledge Levels of Students in a Public Primary School,” International Journal of Caring Sciences 10, no. 1 (2017): 479–486, https://www.proquest.com/docview/1896832951/fulltextPDF?accountid=36155&pq‐origsite=primo&searchKeywords=Arikan%20D%2C%20Alpugan%20N%2C%20Kili%C3%A7%20A.%20Investigation%20of%20personal%20hygiene%20habits%20and%20knowledge%20levels%20of%20students%20in%20a%20public%20primary%20school.%20International%20Journal%20of%20Caring%20Sciences.%202017%3B10(1)%3A479%E2%80%93486.&sourcetype=Scholarly%20Journals.

[16] E. Azor‐Martinez , E. Cobos‐Carrascosa , F. Gimenez‐Sanchez , et al., “Effectiveness of a Multifactorial Handwashing Program to Reduce School Absenteeism due to Acute Gastroenteritis,” American Journal of Infection Control 44, no. 8 (2016): e145–e149, 10.1016/j.ajic.2016.03.014. 24096730

[17] E. Azor‐Martínez , Y. González‐Jiménez , M. L. Seijas‐Vázquez , et al., “The Impact of Common Infections on School Absenteeism During an Academic Year in Primary School Children,” BMC Public Health 16 (2016): 727, 10.1186/s12889-016-3402-9. 24837113

[18] D. Biswas , M. Roy , and A. Pal , “Impact of School Hygiene Education Program on Knowledge and Practice Among School Children in Urban Slums of India,” International Journal of Community Medicine and Public Health 7, no. 11 (2020): 4452–4458, https://uws.primo.exlibrisgroup.com/permalink/61UWSTSYD_INST/1hi497g/cdi_crossref_primary_10_18203_2394_6040_ijcmph20172846.

[19] H. Vally , C. McMichael , C. Doherty , X. Li , and G. Guevarra , “The Impact of a School‐Based Water, Sanitation and Hygiene Intervention on Knowledge, Practices and Diarrhoea Rates in The Philippines,” Environmental Science and Pollution Research 26, no. 35 (2019): 35469–35478, https://www.proquest.com/docview/2329652707. 10.3390/ijerph16214056 PMC6861971 31652683

[20] A. G. Tunio and J. Ahmed , “Impact of Hand Hygiene Interventions on Handwashing Practices and Microbial Risk: A Study in an Orphanage‐Based School in Pakistan,” American Journal of Infection Control 53, no. 2 (2025): 218–224, 10.1016/j.ajic.2024.09.008. 39278264

[21] B. A. Caruso , M. C. Freeman , J. V. Garn , et al., “Assessing the Impact of a School‐Based Latrine Cleaning and Handwashing Program on Pupil Absence in Nyanza Province, Kenya: A Cluster‐Randomized Trial,” Tropical Medicine & International Health 19, no. 3 (2014): 412–427, 10.1111/tmi.12360. PMC4876949 25055716

[22] V. Trinies , J. V. Garn , H. H. Chang , et al., “The Impact of a School‐Based Water, Sanitation, and Hygiene Program on Health and Education Outcomes in Rural Kenya: A Cluster‐Randomized Controlled Trial,” American Journal of Tropical Medicine and Hygiene 94, no. 6 (2016): 1418–1425, 10.4269/ajtmh.15-0757. 27114292 PMC4889767

[23] J. A. Nicholson , M. Naeeni , M. Hoptroff , et al., “An Investigation of the Effects of a Hand Washing Intervention on Health Outcomes and School Absence Using a Randomised Trial in Indian Urban Communities,” Tropical Medicine & International Health 19, no. 3 (2014): 284–292, 10.1111/tmi.12254. 24382344

[24] P. P. L. Or , P. T. Y. Ching , and J. W. Y. Chung , “A Program to Improve the Hand Hygiene Compliance of Hong Kong Preschoolers With an Insight Into Their Absenteeism,” American Journal of Infection Control 47, no. 5 (2019): 498–503, 10.1016/j.ajic.2018.11.014. 30612818

[25] P. P. L. Or , P. T. Y. Ching , and J. W. Y. Chung , “Can Flu‐Like Absenteeism in Kindergartens Be Reduced Through Hand Hygiene Training for Both Parents and Their Kindergarteners?,” Journal of Primary Care & Community Health 11 (2020): 2150132719901209, 10.1177/2150132719901209. PMC6970472 31948327

[26] T. H. Koep , S. Jenkins , M. M. Hammerlund , et al., “Promotion of Influenza Prevention Beliefs and Behaviors Through Primary School Science Education,” Journal of Community Medicine & Health Education 6, no. 3 (2016): 444, https://www.ncbi.nlm.nih.gov/pmc/articles/PMC4982516/. 27525193 10.4172/2161-0711.1000444 PMC4982516

[27] R. Cañete , Y. Campos , R. Valdes , and P. Rodriguez , “Prevalence and Factors Associated With Intestinal Parasitic Infection Among Schoolchildren From Jagüey Grande Municipality in Matanzas Province, Cuba,” West Indian Medical Journal 66, no. 2 (2017): 361–366, https://www.mona.uwi.edu/fms/wimj/system/files/article_pdfs/wimj‐iss2‐2017_361_366_0.pdf.

[28] W. Elmonir , H. Elaadli , A. Amer , et al., “Prevalence of Intestinal Parasitic Infections and Their Associated Risk Factors Among Preschool and School Children in Egypt,” PLoS One 16, no. 9 (2021): e0258037, https://journals.plos.org/plosone/article?id=10.1371/journal.pone.0258037. 34587187 10.1371/journal.pone.0258037 PMC8480785

[29] G. B. Roro , F. Eriso , A. M. Al‐Hazimi , et al., “Prevalence and Associated Risk Factors of *Entamoeba histolytica* Infection Among School Children From Three Primary Schools in Arsi Town, West Zone, Ethiopia,” Journal of Parasitology and Diseases 46, no. 3 (2022): 776–784, 10.1007/s12639-022-01495-1. PMC9458809 36091282

[30] M. A. Mahmud , M. Spigt , A. M. Bezabih , I. L. Pavon , G. J. Dinant , and R. B. Velasco , “Efficacy of Handwashing With Soap and Nail Clipping on Intestinal Parasitic Infections in School‐Aged Children: A Factorial Cluster Randomized Controlled Trial,” PLoS Medicine 12, no. 6 (2015): e1001837, 10.1371/journal.pmed.1001837. 26057703 PMC4461173

[31] A. A. Kim and T. Wu , “Assessment of Diarrheal Rates in a Population of Children in the Indian Himalayas: A Student Initiative,” Annals of Global Health 81, no. 1 (2015): 224, https://annalsofglobalhealth.org/articles/2023/files/submission/proof/2023‐1‐4153‐1‐10‐20180711.pdf.

[32] A. M. Denbæk , A. Andersen , C. T. Bonnesen , et al., “Effect Evaluation of a Randomized Trial to Reduce Infectious Illness and Illness‐Related Absenteeism Among Schoolchildren: The Hi Five Study,” Pediatric Infectious Disease Journal 37, no. 1 (2018): 16–21, https://journals.lww.com/pidj/abstract/2018/01000/effect_evaluation_of_a_randomized_trial_to_reduce.3.aspx. 28746262 10.1097/INF.0000000000001686

[33] L. Borch , K. Thorsteinsson , T. C. Warner , et al., “COVID‐19 Reopening Causes High Risk of Irritant Contact Dermatitis in Children,” Danish Medical Journal 67, no. 9 (2020): A05200357, https://vbn.aau.dk/en/publications/covid‐19‐reopening‐causes‐high‐risk‐of‐irritant‐contact‐dermatiti. 32800064

[34] S. M. Hantoosh , “Hand Hygiene and Water Quality Assessment in Schools of Muthanna Province, Southern Iraq,” Journal of Infection in Developing Countries 17, no. 4 (2023): 518–524, https://pubmed.ncbi.nlm.nih.gov/37159893/. 37159893 10.3855/jidc.17264

[35] P. Priest , J. E. McKenzie , R. Audas , M. Poore , and C. Brunton , “Hand Sanitiser Provision for Reducing Illness Absences in Primary School Children: A Cluster Randomised Trial,” PLoS Medicine 11, no. 8 (2014): e1001700, 10.1371/journal.pmed.1001700. 25117155 PMC4130492

[36] M. C. Freeman , T. Clasen , R. Dreibelbis , et al., “The Impact of a School‐Based Water Supply and Treatment, Hygiene, and Sanitation Programme on Pupil Diarrhoea: A Cluster‐Randomized Trial,” Epidemiology and Infection 142, no. 2 (2014): 340–351, https://www.cambridge.org/core/journals/epidemiology‐and‐infection/article/impact‐of‐a‐schoolbased‐water‐supply‐and‐treatment‐hygiene‐and‐sanitation‐programme‐on‐pupil‐diarrhoea‐a‐clusterrandomized‐trial/43B32A94B5DD320C19D133F23A90F2D4. 23702047 10.1017/S0950268813001118 PMC9151148

[37] Y. Otsuka , L. Agestika , H. Harada , L. Sriwuryandari , N. Sintawardani , and T. Yamauchi , “Comprehensive Assessment of Handwashing and Faecal Contamination Among Elementary School Children in an Urban Slum of Indonesia,” Tropical Medicine & International Health 24, no. 8 (2019): 954–961, 10.1111/tmi.13279. 31192489

[38] S. K. Padaruth and S. D. Biranjia‐Hurdoyal , “Hygiene Practices and Faecal Contamination of the Hands of Children Attending Primary School in Mauritius,” International Health 7, no. 4 (2015): 280–284, https://academic.oup.com/inthealth/article/7/4/280/2458773?login=false. 25424722 10.1093/inthealth/ihu080

[39] E. Kavitha , G. Malarvizhi , and R. Ganesan , “Bacteriological Profile and Perception on Hand Hygiene Among School Children in Tamil Nadu, India,” International Journal of Community Medicine and Public Health 8, no. 3 (2021): 1147–1152, 10.18203/2394-6040.ijcmph20210788.

[40] K. Klar , D. Knaack , S. Kampmeier , et al., “Knowledge About Hand Hygiene and Related Infectious Disease Awareness Among Primary School Children in Germany,” Children (Basel) 9, no. 2 (2022): 190, https://www.mdpi.com/2227‐9067/9/2/190. 35204911 10.3390/children9020190 PMC8870042

[41] Y. E. Cha , Y. Z. Fu , and W. Yao , “Knowledge, Practice of Personal Hygiene, School Sanitation, and Risk Factors of Contracting Diarrhea Among Rural Students From Five Western Provinces in China,” International Journal of Environmental Research and Public Health 18, no. 18 (2021): 9505, https://www.mdpi.com/1660‐4601/18/18/9505. 34574432 10.3390/ijerph18189505 PMC8468795

[42] E. Azor‐Martínez , Y. Gonzalez‐Jimenez , M. L. Seijas‐Vazquez , et al., “The Impact of Common Infections on School Absenteeism During an Academic Year,” American Journal of Infection Control 42, no. 6 (2014): 632–637, https://www.sciencedirect.com/science/article/pii/S0196655314001333?casa_token=tRT7HY7ibPsAAAAA:c5onKsDv6vkOoqVar6Hmj‐2RXJTdfzrntvSU2GOjdCyyX84qsuKx4vQ‐2ql5zkcYx2CTW092UHGj. 24837113 10.1016/j.ajic.2014.02.017

[43] A. Endo , M. Uchida , N. Hayashi , et al., “Within and Between Classroom Transmission Patterns of Seasonal Influenza Among Primary School Students in Matsumoto City, Japan,” Proceedings of the National Academy of Sciences of the United States of America 118, no. 46 (2021): e2112605118, https://www.pnas.org/doi/abs/10.1073/pnas.2112605118. 34753823 10.1073/pnas.2112605118 PMC8609560

[44] A. Matsuda , K. Asayama , T. Obara , N. Yagi , and T. Ohkubo , “Behavioral Changes of Preventive Activities of Influenza Among Children in Satellite Cities of a Metropolitan Area of Tokyo, Japan, by the COVID‐19 Pandemic,” BMC Public Health 23, no. 1 (2023): 727, 10.1186/s12889-023-15629-2. 37085782 PMC10119014

[45] N. Torner Gràcia , N. Soldevila , J. J. Garcia , et al., “Effectiveness of Non‐Pharmaceutical Measures in Preventing Pediatric Influenza: A Case–Control Study [Internet],” https://repositori.udl.cat/server/api/core/bitstreams/623d74c8‐1340‐42d8‐ba62‐ec63748dcb2e/content. 10.1186/s12889-015-1890-3 PMC4459072 26055522

[46] K. Riiser , S. Helseth , K. Haraldstad , A. Torbjørnsen , and K. R. Richardsen , “Adolescents' Health Literacy, Health Protective Measures, and Health‐Related Quality of Life During the COVID‐19 Pandemic,” PLoS One 15, no. 8 (2020): e0238161, https://journals.plos.org/plosone/article?id=10.1371/journal.pone.0238161. 32857806 10.1371/journal.pone.0238161 PMC7454983

[47] M. C. Freeman , L. E. Greene , R. Dreibelbis , et al., “Assessing the Impact of a School‐Based Water Treatment, Hygiene and Sanitation Programme on Pupil Absence in Nyanza Province, Kenya: A Cluster‐Randomized Trial,” Tropical Medicine & International Health 17, no. 3 (2012): 380–391, 10.1111/j.1365-3156.2011.02927.x. 22175695

[48] A. A. Alzaher , S. S. Almudarra , M. H. Mustafa , and I. M. Gosadi , “The Importance of Hand Hygiene Education on Primary Schoolgirls' Absence due to Upper Respiratory Infections in Saudi Arabia: A Cluster Randomized Controlled Trial,” Saudi Medical Journal 39, no. 10 (2018): 1044–1049, https://pmc.ncbi.nlm.nih.gov/articles/PMC6201029/. 30284589 10.15537/smj.2018.10.23344 PMC6201029

[49] V. Curtis and S. Cairncross , “Effect of Washing Hands With Soap on Diarrhoea Risk in the Community: A Systematic Review,” Lancet Infectious Diseases 3, no. 5 (2003): 275–281, 10.1016/S1473-3099(03)00606-6. 12726975

[50] A. E. Aiello , R. M. Coulborn , V. Perez , and E. L. Larson , “Effect of Hand Hygiene on Infectious Disease Risk in the Community Setting: A Meta‐Analysis,” American Journal of Public Health 98, no. 8 (2008): 1372–1381, 10.2105/AJPH.2007.124610. 18556606 PMC2446461

[51] C. Jasper , T. T. Le , and J. Bartram , “Water and Sanitation in Schools: A Systematic Review of the Health and Educational Outcomes,” International Journal of Environmental Research and Public Health 9, no. 8 (2012): 2772–2787, 10.3390/ijerph9082772. 23066396 PMC3447586

[52] M. C. Freeman , M. E. Stocks , O. Cumming , et al., “Hygiene and Health: Systematic Review of Handwashing Practices Worldwide and Update of Health Effects,” Tropical Medicine & International Health 19, no. 8 (2014): 906–916, 10.1111/tmi.12339. 24889816

[53] FGPS Challenge , “WHO Guidelines on Hand Hygiene in Health Care,” https://organesdeconcertation.sante.belgique.be/sites/default/files/documents/who_guidelines_on_hand_hygiene_in_health_care.pdf.

[54] A. Joshi and C. Amadi , “Impact of Water, Sanitation, and Hygiene Interventions on Improving Health Outcomes Among School Children,” Journal of Environmental and Public Health 2013 (2013): 984626, 10.1155/2013/984626. 24454415 PMC3888759

[55] G. Hutton , L. Haller , and J. Bartram , “Global Cost‐Benefit Analysis of Water Supply and Sanitation Interventions,” Journal of Water and Health 5, no. 4 (2007): 481–502, 10.2166/wh.2007.009. 17878562

[56] A. Silburn and N. Singh , “Hand Hygiene Practices in Paediatric Populations: Assessing Their Impact on Infectious Disease Outbreaks in Preschools and Schools,” medRxiv (2025), 10.1101/2025.07.02.25330765. PMC12976192 41664625

