# Supplementary material for: Hand Hygiene Practices in School Populations: Assessing Their Impact on Infectious Disease Outbreaks
Source: J Paediatr Child Health. 2026 Feb 10;62(3):334–54. doi: 10.1111/jpc.70311 (PMC12976192; doi:10.1111/jpc.70311)
Supplement: Supplementary file 1 — Appendix S1: Hand hygiene practices in paediatric populations: Search strategy. This appendix provides the complete search strategy used to identify studies on hand hygiene practices and interventions in paediatric populations. It includes database names, search dates, search strings, Boolean operators and any applied filters or language restrictions. [file JPC-62-334-s002.docx]

Hand Hygiene Practices in Paediatric Populations: Assessing Their Impact on Infectious Disease Outbreaks in Preschools and Schools.

**Search Strategy**

A literature search will be conducted in the electronic databases MEDLINE Complete, Embase, and PubMed, as they have proven worth in producing credible literature covering a wide range of scientific, medical, and healthcare disciplines. In addition, the Education Resources Information Center (ERIC) electronic database will permit literature from the educational perspective of disease burden, thus adequate for the proposed research question.

To generate a valid script, the terms needed to be contemplated to ensure the results were relevant whilst not being over-limited. In addition, terminology variation was considered to permit international applications. To achieve this, truncation methods and Boolean operators were applied as a strategic means to produce relevant results for possible inclusion. The keywords or MeSH (Medical Subject Headings) to be used in the search are ‘respiratory tract infection, common cold, influenza, Coronavirus, respiratory syncytial virus, viral gastroenteritis, salmonella, campylobacter, Escherichia coli, shigella, staphylococcus aureus’. Population-defining terms used will be ‘student, school, preschool, daycare, child, children, or infants’. For the intervention, proximity operators will be utilised within seven words between ‘hand’ and ‘wash, disinfect, sanitise, clean, or hygiene’.

This resulted in the following action:

***(MeSH: respiratory tract infection OR common cold OR influenza OR Coronavirus OR respiratory syncytial virus OR Viral gastroenteritis OR salmonella OR campylobacter OR Escherichia coli OR shigella OR staphylococcus aureus)***

***AND***

***(student OR school OR preschool OR daycare OR child OR children OR infants)***

***AND***

***(hand adj7 (wash* OR disinfect* OR saniti* OR clean* OR hygiene))***

The following demonstrates the Search Strategy contextualised to each database.

**Medline and Embase:**

| ***#*** | ***Query*** |
| --- | --- |
| *1* | *Respiratory Tract Infections/* |
| *2* | *Common Cold/* |
| *3* | *Influenza, Human/* |
| *4* | *Coronavirus/* |
| *5* | *Respiratory Syncytial Viruses/* |
| *6* | *Virus Diseases/ or Rotavirus/ or Norovirus/ or Diarrhea/ or Gastroenteritis/ or Rotavirus Infections/* |
| *7* | *Salmonella/* |
| *8* | *Campylobacter/* |
| *9* | *Escherichia coli/* |
| *10* | *Shigella/* |
| *11* | *Staphylococcus aureus/* |
| *12* | *1 or 2 or 3 or 4 or 5 or 6 or 7 or 8 or 9 or 10 or 11* |
| *13* | *(student or school or preschool or daycare or child or children or infants).mp. [mp=title, book title, abstract, original title, name of substance word, subject heading word, floating sub-heading word, keyword heading word, organism supplementary concept word, protocol supplementary concept word, rare disease supplementary concept word, unique identifier, synonyms, population supplementary concept word, anatomy supplementary concept word]* |
| *14* | *(hand adj7 (wash* or disinfect* or saniti* or clean* or hygiene)).mp. [mp=title, book title, abstract, original title, name of substance word, subject heading word, floating sub-heading word, keyword heading word, organism supplementary concept word, protocol supplementary concept word, rare disease supplementary concept word, unique identifier, synonyms, population supplementary concept word, anatomy supplementary concept word]* |
| *15* | *12 and 13 and 14* |

**PubMed:**

*(("respiratory tract infection"[All Fields] OR "common cold"[All Fields] OR "influenza"[All Fields] OR "coronavirus"[All Fields] OR "respiratory syncytial virus"[All Fields] OR "viral gastroenteritis"[All Fields] OR "salmonella"[All Fields] OR "campylobacter"[All Fields] OR "escherichia coli"[All Fields] OR "shigella"[All Fields] OR "staphylococcus aureus"[All Fields]) AND ("student s"[All Fields] OR "students"[MeSH Terms] OR "students"[All Fields] OR "student"[All Fields] OR "students s"[All Fields] OR ("educational status"[MeSH Terms] OR ("educational"[All Fields] AND "status"[All Fields]) OR "educational status"[All Fields] OR "schooling"[All Fields] OR "education"[MeSH Terms] OR "education"[All Fields] OR "school s"[All Fields] OR "schooled"[All Fields] OR "schools"[MeSH Terms] OR "schools"[All Fields] OR "school"[All Fields]) OR ("child, preschool"[MeSH Terms] OR ("child"[All Fields] AND "preschool"[All Fields]) OR "preschool child"[All Fields] OR "preschooler"[All Fields] OR "preschoolers"[All Fields] OR "preschool"[All Fields] OR "preschooler s"[All Fields] OR "preschools"[All Fields]) OR ("child day care centers"[MeSH Terms] OR ("child"[All Fields] AND "day"[All Fields] AND "care"[All Fields] AND "centers"[All Fields]) OR "child day care centers"[All Fields] OR "daycare"[All Fields] OR "daycares"[All Fields]) OR ("child"[MeSH Terms] OR "child"[All Fields] OR "children"[All Fields] OR "child s"[All Fields] OR "children s"[All Fields] OR "childrens"[All Fields] OR "childs"[All Fields]) OR ("child"[MeSH Terms] OR "child"[All Fields] OR "children"[All Fields] OR "child s"[All Fields] OR "children s"[All Fields] OR "childrens"[All Fields] OR "childs"[All Fields]) OR ("infant"[MeSH Terms] OR "infant"[All Fields] OR "infants"[All Fields] OR "infant s"[All Fields])) AND (("hand"[MeSH Terms] OR "hand"[All Fields]) AND ("wash*"[All Fields] OR "disinfect*"[All Fields] OR "saniti*"[All Fields] OR "clean*"[All Fields] OR ("hygiene"[MeSH Terms] OR "hygiene"[All Fields] OR "hygienic"[All Fields] OR "hygienical"[All Fields] OR "hygienically"[All Fields] OR "hygienics"[All Fields] OR "hygienization"[All Fields]))))*

**ERIC:**

*(“respiratory tract infection” OR “common cold” OR influenza OR Coronavirus OR “respiratory syncytial virus” OR “Viral gastroenteritis” OR salmonella OR campylobacter OR “Escherichia coli” OR shigella OR “staphylococcus aureus”) AND (student OR school OR preschool OR daycare OR child OR children OR infants) AND (hand NEAR/7 (wash* OR disinfect* OR saniti* OR clean* OR hygiene))*
